# Self-reported strengths and difficulties among Swedish adolescents: presence, continuity and change

**DOI:** 10.1093/eurpub/ckac131.464

**Published:** 2022-10-25

**Authors:** K Grigoryan, V Östberg, J Raninen, J Åhlén, S B Låftman

**Affiliations:** 1 Department of Public Health Sciences, Stockholm University, Stockholm, Sweden; 2 Department of Clinical Neuroscience, Karolinska Institutet, Stockholm, Sweden; 3 Centre for Alcohol Policy Research, La Trobe University, Melbourne, Australia; 4 Centre for Epidemiology & Community Medicine, Stockholm County Council, Stockholm, Sweden; 5 Department of Global Public Health, Karolinska Institutet, Stockholm, Sweden

## Abstract

**Background:**

The Strengths and Difficulties Questionnaire (SDQ) is a screening instrument for emotional and behavioural problems. Swedish reference values based on large-scale national data are lacking for mid- and late adolescents. Also, there is a scarcity of longitudinal studies about the development of strengths and difficulties from middle to late adolescence. The study aims were 1) to report on Swedish adolescents’ assessment of their strengths and difficulties and to present joint and gender-specific reference values for identifying risk groups; 2) to examine the continuity and change of strengths and difficulties and to what extent this differs by gender.

**Methods:**

Data was based on a national Swedish sample of adolescents aged 15-16 years in 2017 (n = 5338) who were surveyed again in 2019 (n = 3973). Mean values and reference values for “close to average” (0th-80th percentile), “slightly raised” (>80th-90th percentile) and “high” (>90th percentile) SDQ scores were calculated. Comparison in SDQ scores by gender and across time was approached using inferential statistics.

**Results:**

Girls reported higher levels of emotional problems (p = <0.001), whereas boys showed higher levels of conduct problems (p = <0.001) at t1 and t2. Hyperactivity slightly prevailed in girls (p = 0.007), and peer problems were slightly higher in boys (p = 0.027) at t2. Prosocial behaviour was higher in girls than boys (p = <0.001) at t1 and t2. Changes in SDQ scores across time were in general small. Yet, analyses focusing on risk groups showed that among those who scored >80th or > 90th percentile at t1 about half scored above the same threshold at t2.

**Conclusions:**

This study provided joint and gender-specific reference values for mid- and late adolescents in Sweden, and found some gender differences in SDQ scores and degree of change. Reported national gender-specific reference values may facilitate identifying adolescents at risk and potentially increasing timely mental health intervention measures.

**Key messages:**

• Among the subscales indicating difficulties, the highest was level of emotional problems in girls and hyperactivity in boys; the largest increase over time was in emotional problems for both genders.

• Analyses of changes in SDQ mean scores showed only minor changes between t1 and t2, while analyses of movement between categories defined by cutoffs presented a more noticeable degree of change.

